# Subcutaneous extensor tendon rupture caused by Kienböck disease complicated by carpal tunnel syndrome: A case report

**DOI:** 10.1097/MD.0000000000047001

**Published:** 2026-01-09

**Authors:** Yoshiaki Tomizuka, Soya Nagao, Koji Tanimoto, Kana Okugawa, Hiroko Shiraishi, Tomonori Kinoshita, Shunsuke Ueno, Kazuyoshi Nakanishi

**Affiliations:** a Department of Orthopaedic Surgery, Nihon University School of Medicine, Tokyo, Japan; b Department of Orthopaedics and Rehabilitation, Itabashi Medical Association Hospital, Tokyo, Japan.

**Keywords:** carpal tunnel syndrome, case report, extensor tendon rupture, Kienböck disease

## Abstract

**Rationale:**

Subcutaneous extensor tendon rupture caused by Kienböck disease is rare. Only 22 cases have been reported in the English literature since 1986.

**Patient concerns:**

A 74-year-old male experienced numbness of his right hand for several years and was unable to extend his right middle and ring finger 2 months before consultation.

**Diagnoses:**

Physical examination revealed no pain in his right wrist, but was unable to extend the middle and ring fingers. He also had numbness of the thumb, index, middle, and radial side of the ring finger. Plain radiography and computed tomography revealed osteoarthritis of the wrist and segmental lunate bone. Ultrasonography revealed disruption of the extensor tendon, indicating that the lunate bone volar fragment did not interfere with the flexor tendons and median nerve. Magnetic resonance imaging suggested extensor tendon rupture. Nerve conduction studies showed delayed distal motor latencies of the abductor pollicis brevis muscle. We diagnosed subcutaneous rupture of the extensor tendons of the middle and ring fingers caused by stage Ⅳ Kienböck disease complicated by carpal tunnel syndrome.

**Interventions:**

A 2-portal endoscopic carpal tunnel release was performed under general anesthesia. A dorsal curved incision was made. The dorsal fragment of the lunate punctured the capsule, and the extensor digitorum communis (EDC) tendon of the middle, ring, and little fingers, and the extensor indicis proprius tendons were ruptured. The dorsal fragment of the lunate bone was removed and the EDC tendon of the middle finger was transferred to that of the index finger. The combined EDC tendons of the ring and little fingers were transferred to the extensor digitorum minimi tendon.

**Outcomes:**

The numbness and extension restriction of the middle and ring fingers had improved at 2 years postoperatively. Dorsal and volar flexion were up to 60°.

**Lessons:**

Dorsal lunate bone fragments associated with advanced Kienböck disease can cause extensor tendon rupture. When ultrasonography confirms that the volar fragment of the lunate bone does not impinge upon the median nerve or flexor tendons, surgical intervention on the volar fragment may be unnecessary. Computed tomography, magnetic resonance imaging, and ultrasonography are valuable for accurate assessment and preoperative planning.

## 1. Introduction

Rupture of the subcutaneous extensor tendon of the fingers is common in patients with rheumatoid arthritis or osteoarthritis. However, attritional extensor tendon rupture caused by Kienböck disease is rare, and only 22 cases have been reported in the English literature since 1986.^[1–11]^ Furthermore, only 1 case presenting with carpal tunnel syndrome has been reported.^[2]^ Therefore, no consensus on the treatment of extensor tendon rupture or carpal tunnel syndrome associated with Kienböck disease has been established. We present a case of attritional rupture of the extensor digitorum communis (EDC) tendon of the middle, ring, and little fingers, and the extensor indicis proprius (EIP) tendons caused by Kienböck disease complicated by carpal tunnel syndrome.

## 2. Case report

**Figure 1.**
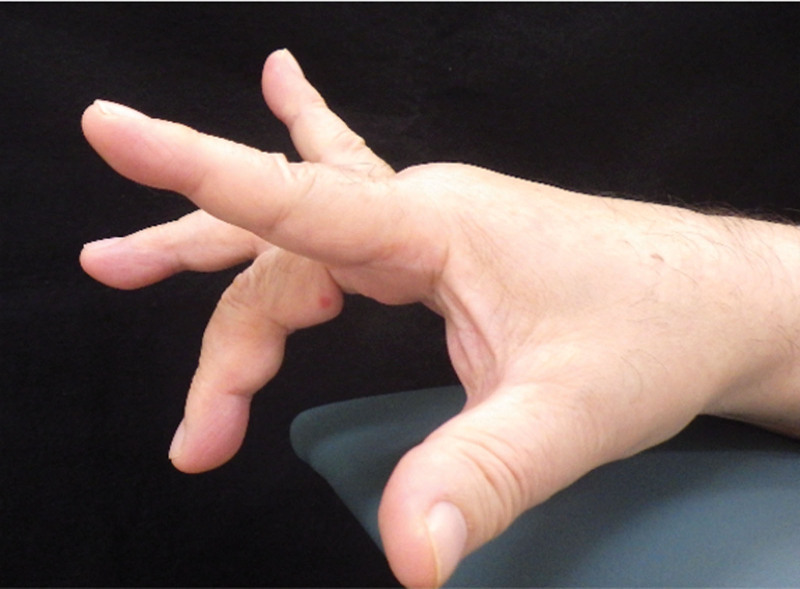
Clinical findings at presentation. The patient could not extend the right middle and ring finger.

**Figure 2.**
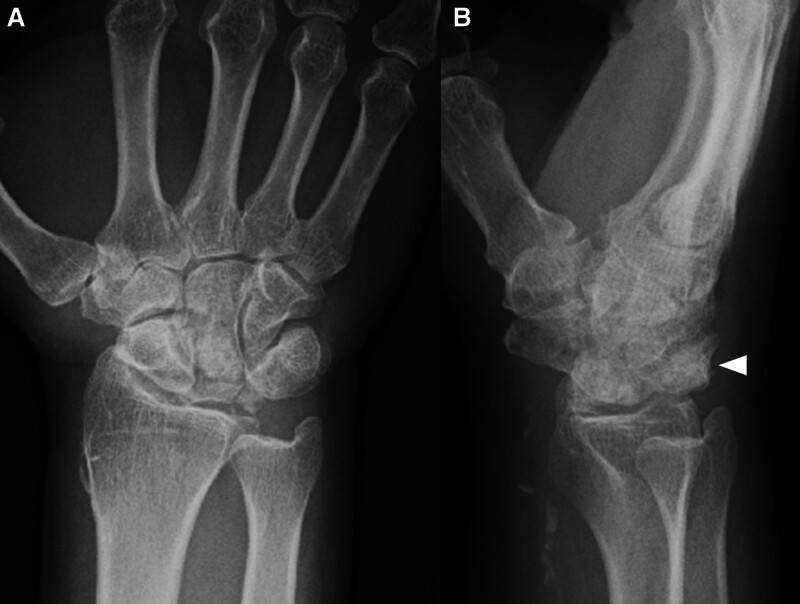
Initial plain radiographs. (A) Anteroposterior and (B) lateral view indicate osteoarthritis of wrist and segmented lunate bone (arrowhead).

**Figure 3.**
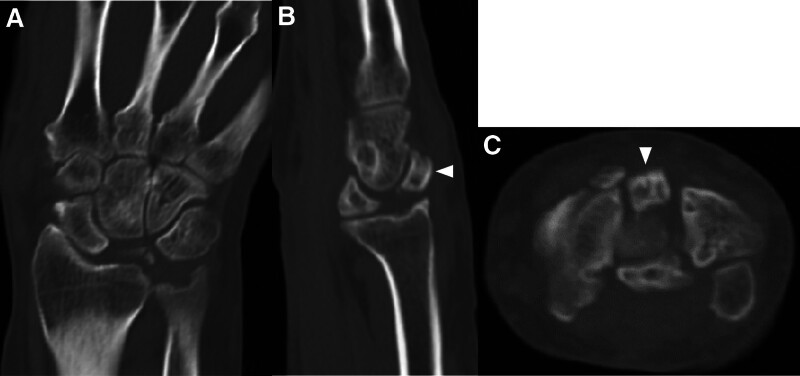
Initial plain computed tomography. (A) Coronal, (B) sagittal, and (C) axial view of right wrist showing displaced volar and dorsal segment of lunate bone (arrowhead).

**Figure 4.**
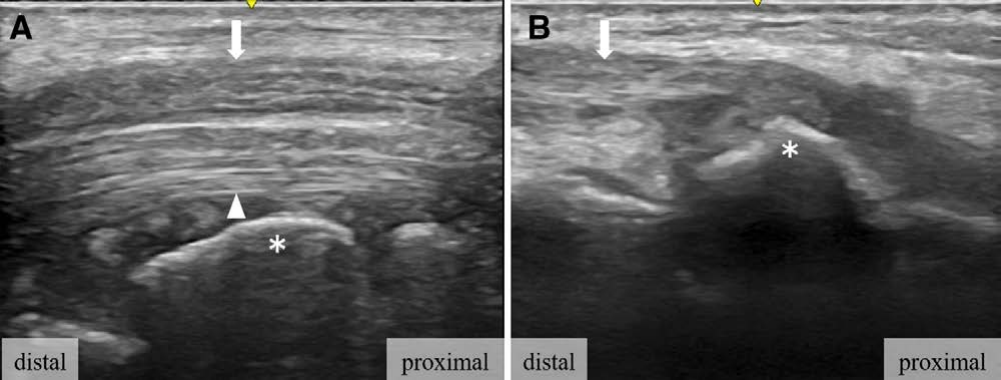
Ultrasound findings. (A) Longitudinal view to the volar aspect of wrist showing volar segment of lunate bone (asterisk) that does not interfere with the median nerve (arrow) or flexor tendon (arrowhead). (B) Longitudinal view to the dorsal aspect of wrist showing distal stump of ruptured extensor tendon (arrow) and dorsal segment of lunate bone (asterisk).

**Figure 5.**
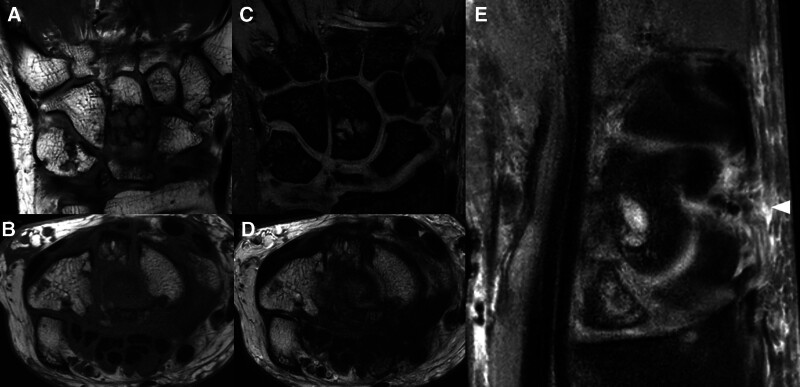
MRI. T1-weighted images on (A) the coronal and (B) axial view indicating the lunate bone as a low-intensity area. T2-weighted images on (C) the coronal and (D) axial view indicating the lunate bone as a partially high-intensity area. (E) The extensor tendon is not clear on sagittal T2-weighted images, which suggests rupture of the extensor tendon. MRI = magnetic resonance imaging.

**Figure 6.**
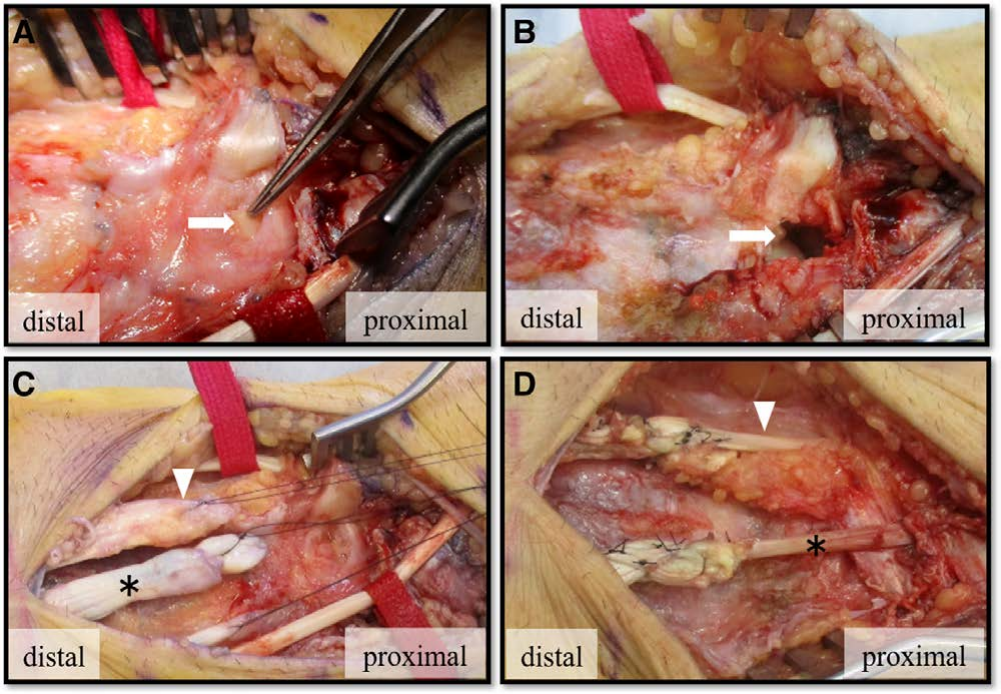
Operative findings of dorsal aspect of the wrist. (A) EDC tendon of the middle, ring, and little finger and EIP tendon were ruptured at the site the lunate bone punctured the dorsal capsule (arrow). (B) The dorsal segment of lunate bone was removed (arrow). (C) Combined distal end of the EDC tendon of the middle and ring finger (arrowhead) and the EDC tendon of the index finger (asterisk) were isolated. (D) The EDC tendon of the middle finger was transferred to the EDC tendon of the index finger (asterisk). The combined suture of the EDC tendon of ring and little finger were transferred to the extensor digit minimi tendon (arrowhead). The distal stump of EIP tendon was excised. EDC = extensor digitorum communis, EIP = extensor indicis proprius.

**Figure 7.**
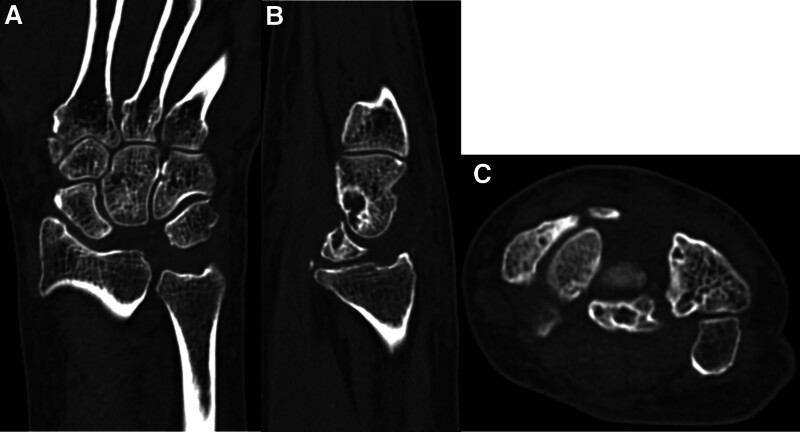
Plain computed tomography at 6 mo after surgery. (A) Coronal, (B) sagittal, and (C) axial view of right wrist indicating the dorsal fragment of the lunate bone was removed, and the volar fragment was retained.

**Figure 8.**
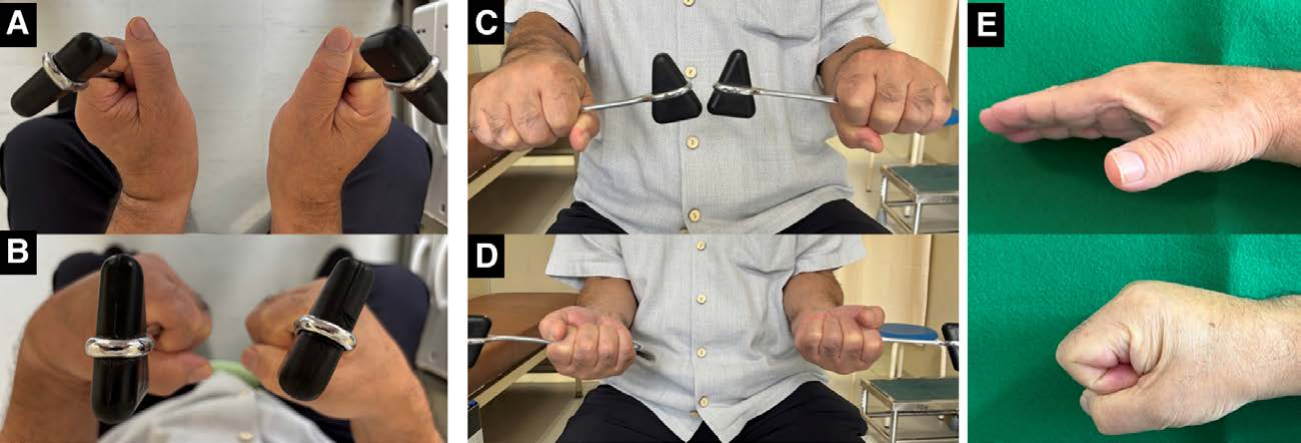
Clinical findings at 2 yr after surgery. Each of (A) dorsal and (B) volar flexion of the wrist were up to 60°. The forearm could be pronated (C) and supinated (D) to 90°. (E) Finger motion was improved.

A 74-year-old male experienced numbness in his right hand for several years and noticed that he was unable to extend his right middle and ring fingers while bathing 2 months before presenting for consultation. The patient was referred to our hospital for further treatment. He had a past medical history of pemphigus, hypertension, and hyperlipidemia. He had a history of prednisolone use. Physical examination revealed restriction of his right wrist motion; dorsal flexion of 45°; volar flexion of 60°; numbness of the right thumb, index, middle, and radial sides of the ring finger; and inability to extend the right middle and ring fingers (Fig. 1). Tenodesis effect of his middle and ring fingers was absent, and he had no right wrist pain. In addition, Tinel sign and Phalen test results were positive, but thenar atrophy was absent. His grip strength was 29.7 kg. Plain radiography (Fig. 2A and B) and computed tomography (CT) (Fig. 3A–C) showed a segmented lunate bone and a dorsal fragment of the lunate bone protruding with osteoarthritis of the midcarpal and radiocarpal joints. Ultrasound examination revealed that the volar fragment of the lunate bone did not interfere with the flexor tendons or median nerve, but disruption of the extensor tendon was observed (Fig. 4A and B). Magnetic resonance imaging revealed that the lunate bone appeared as a low-intensity area on T1-weighted images and as a partially high-intensity area on T2-weighted images. Additionally, the extensor tendon was poorly visualized on sagittal T2-weighted images, suggesting its rupture (Fig. 5A–E). Nerve conduction studies showed a delay in distal motor latencies (5.3 ms), although an active potential of the sensory nerve of the abductor pollicis brevis muscle could not be detected (Bland classification Grade 4). Subcutaneous extensor tendon rupture caused by Lichtman classification grade Ⅳ Kienböck disease complicated by carpal tunnel syndrome was diagnosed, and surgery was planned. Preoperatively, the quick DASH score was 13.7 points, the scores of the symptom and functional scales of Boston Carpal Tunnel Questionnaire were 1.8 and 1.6 points, respectively. Informed consent was obtained before surgery. He also provided written informed consent for the use and publication of his anonymized diagnostic images, clinical photographs, and other case-related data. Surgery was performed 2 months after the onset of symptoms for a diagnosis of carpal tunnel syndrome combined with subcutaneous rupture of the extensor tendon caused by Kienböck disease under general anesthesia. We first performed a 2-portal endoscopic carpal tunnel release (ECTR). Only ECTR was performed on the volar side, but the volar fragment of the lunate bone was not treated. Following this, a curved dorsal incision was made. The dorsal fragment of the lunate bone punctured the dorsal capsule, and at this site, the EDC tendons of the middle, ring, and little fingers, as well as the EIP tendon, were ruptured. The dorsal fragment of the lunate bone was removed, and the middle finger EDC tendon was transferred to the index finger EDC tendon. A combined suture of the EDC tendons of the ring and little fingers was transferred to the extensor digitorum minimi tendon (Fig. 6A–D). The distal stump of the EIP tendon was excised. His right wrist was externally fixed with dorsiflexion for 3 weeks after surgery, and he was permitted active motion of the wrist, but the fingers were treated with taping. Plain CT at 6 months after surgery showed that the dorsal fragment of the lunate bone had been removed (Fig. 7A–C). Physical examination 2 years after the surgery revealed no pain in his right wrist, and his right finger numbness had improved. His wrist showed 60° dorsal and volar flexion. His finger motion had also improved (Fig. 8A–E) and his grip strength was 31.8 kg. His quick DASH score was 2.2 points, and his scores for the symptom and functional scales of the Boston Carpal Tunnel Questionnaire were 1.1 and 1.0 points, respectively. He reported a high postoperative satisfaction.

## 3. Discussion

Spontaneous extensor tendon rupture is a rare complication of Kienböck disease and was first reported by Miki et al^[1]^ in 1986. Only 22 cases have been reported in the English literature.^[1–11]^ Our case was complicated by carpal tunnel syndrome. However, Kienböck disease has not been established as a cause of carpal tunnel syndrome, and a consensus on the treatment approach for complicated carpal tunnel syndrome is lacking. Choi et al reported a case of closed extensor tendon rupture caused by Kienböck disease complicated by carpal tunnel syndrome.^[2]^ They performed open carpal tunnel release, tendon transfer and excision of dislocated lunate bone. In our case, ultrasonography revealed that the volar fragment of the lunate bone did not interfere with the median nerve or flexor tendons. Extensive incisions on both the volar and dorsal sides of the wrist may lead to more severe postoperative swelling, and ECTR was selected for the treatment of carpal tunnel syndrome. Based on our review of the literature, no previous reports have described the simultaneous performance of dorsal lunate fragment excision and ECTR.

Various reports have described the management of the lunate bone, including procedures such as excision of only the dorsal fragment or complete excision. The volar fragment is attached to the short radiolunate ligament and contributes to the palmar stability of the wrist. Therefore, preservation of the volar fragment of the lunate bone is preferable if removal is unnecessary. Park et al completely excised the lunate bone to eliminate the possibility of flexor tendon rupture and carpal tunnel syndrome and added scaphocapitate (SC) fusion.^[3]^ Tomori et al also completely excised the lunate bone and performed SC fusion; however, they mentioned the possibility of unnecessary arthroplasty and arthrodesis.^[4]^ Turner et al used ultrasonography to confirm that the prominent volar fragment of the lunate bone was in contact with the flexor digitorum profundus tendon, and they excised the volar fragment of the lunate bone.^[12]^ In our case, the volar fragment of the lunate did not interfere with the flexor tendons or median nerve, and excision of the volar fragment of the lunate was considered unnecessary. We consider ultrasonography to be superior to magnetic resonance imaging in this context, as it allows dynamic evaluation of the interactions between bone fragments, tendons, and nerves. Ultrasonography may serve as a valuable tool for determining the appropriate therapeutic approach in patients with Kienböck disease complicated by tendon rupture and carpal tunnel syndrome. However, we believe that caution is warranted regarding the potential occurrence of flexor tendon ruptures.

All previously reported cases involved advanced Kienböck disease; however, surgery was mostly limited to partial or complete excision of the lunate bone. To the best of our knowledge, only 9 of 22 cases presented with wrist pain, and treatment for Kienböck disease was not considered necessary in the absence of symptoms. Further, no additional treatment was performed for most cases. Our patient also presented with no wrist pain before surgery, and the course was favorable following only partial excision of the lunate bone with no need for additional treatment for Kienböck disease.

Long-term steroid use may predispose patients to osteonecrosis and tendon fragility. It remains uncertain whether the Kienböck disease in our case was related to steroid administration. Although the presence of preexisting tendon fragility cannot be confirmed, the site of the extensor tendon rupture corresponded precisely to the protruding dorsal fragment of the lunate, suggesting that the rupture was likely attributable to friction against the dorsal lunate fragment associated with Kienböck disease.

## 4. Conclusion

We described a case of subcutaneous extensor tendon rupture caused by Kienböck disease complicated by carpal tunnel syndrome. Only the dorsal fragment of the lunate bone was excised, whereas the volar fragment was preserved. Ultrasonography revealed that the volar fragment of the lunate bone did not directly interfere with the median nerve or flexor tendons; thus, resection of the volar fragment of the lunate was considered unnecessary. Continuous follow-up is needed for the potential occurrence of flexor tendon rupture due to preserved volar fragments of the lunate bone.

## Acknowledgments

We would like to thank Editage (www.editage.jp) for English language editing.

## Author contributions

**Conceptualization:** Yoshiaki Tomizuka, Koji Tanimoto, Kana Okugawa, Hiroko Shiraishi, Tomonori Kinoshita, Shunsuke Ueno.

**Supervision:** Kazuyoshi Nakanishi.

**Writing – original draft:** Yoshiaki Tomizuka.

**Writing – review & editing:** Yoshiaki Tomizuka, Soya Nagao, Kazuyoshi Nakanishi.

## References

[1] Miki T Yamamuro T Kotoura Y Tsuji T Shimizu K Itakura H. Rupture of the extensor tendons of the fingers: report of three unusual cases. J Bone Joint Surg Am. 1986;68:610–4. 3957988

[2] Choi JY Cha WJ Jung ER Seo BF Jung SN. Closed extensor tendon rupture caused by Kienböck disease: a case report. Arch Plast Surg. 2022;49:76–9. 35086314 10.5999/aps.2021.01522 PMC8795655

[3] Park JW Kim SK Park JH Wang JH Jeon WJ. Multiple extensor tendon ruptures with advanced Kienböck’s disease. J Hand Surg Am. 2007;32:233–5. 17275599 10.1016/j.jhsa.2006.11.002

[4] Tomori Y Nanno M Takai S. Closed rupture of extensor tendon resulting from untreated Kienböck disease: a case report and a review of the literature. Medicine (Baltimore). 2019;98:e16900. 31415435 10.1097/MD.0000000000016900 PMC6831435

[5] Inoué G. Attritional rupture of the extensor tendon due to longstanding Kienböck’s disease. Ann Chir Main Memb Super. 1994;13:135–8. 7521659 10.1016/s0753-9053(05)80386-2

[6] Murase T Ando Y Hiroshima K. Extensor tendon rupture due to Kienböck’s disease. J Hand Surg Br. 1997;22:597–8. 9752912 10.1016/s0266-7681(97)80354-3

[7] Ramkumar S Josty IC Sykes PJ. Severe extensor tendon attrition and multiple tendon ruptures resulting from Kienböck’s disease. Ann Plast Surg. 2000;45:647–50. 11128766 10.1097/00000637-200045060-00014

[8] Pacha-Vicente D Sevilla-Tirado J López-Martínez R Lluch-Bergadà A Mir-Bulló X Llusá-Pérez M. Extensor digiti minimi damage due to longstanding Kienböck’s disease. J Hand Surg Eur Vol. 2007;32:231. 10.1016/J.JHSB.2006.11.012 17222487

[9] Niwa T Uchiyama S Yamazaki H Kasashima T Tsuchikane A Kato H. Closed tendon rupture as a result of Kienböck disease. Scand J Plast Reconstr Surg Hand Surg. 2010;44:59–63. 20367065 10.3109/02844310903351301

[10] Hernández-Cortés P Pajares-López M Gómez-Sánchez R Garrido-Gómez J Lara-García F. Rupture of extensor tendon secondary to previously undiagnosed Kienböck disease. J Plast Surg Hand Surg. 2012;46:291–3. 22747360 10.3109/2000656X.2012.668325

[11] Kim TG Heo YM Min YK. Extensor tendon rupture due to advanced Kienböck’s disease: two case reports and a review of literature. J Hand Surg Asian Pac Vol. 2020;25:123–8. 32000607 10.1142/S2424835520720042

[12] Turner K Sheppard NN Norton SE. Flexor tendon rupture due to previously undiagnosed Kienböck disease: a case report. Hand (N Y). 2017;12:NP37–8. 28453342 10.1177/1558944716668861 PMC5480672

